# Isolation and characterization of *Priestia megaterium* KD7 for the biological control of pear fire blight

**DOI:** 10.3389/fmicb.2023.1099664

**Published:** 2023-03-09

**Authors:** Zeling Cui, Lina Hu, Linglu Zeng, Wanqiu Meng, Dong Guo, Li Sun

**Affiliations:** College of Life Sciences, Shihezi University, Shihezi, Xinjiang, China

**Keywords:** biocontrol, antibacterial activity, *Priestia megaterium*, extracellular metabolites, fire blight

## Abstract

*Erwinia amylovora* is a plant pathogen that causes fire blight disease in Rosaceous plants, such as pear and apple. To develop an effective biocontrol method to suppress *E. amylovora*, a total of 16 bacteria were isolated from pear orchard soil in China and screened for antagonistic activity *in vitro*. Among them, 9 isolates that exhibited antagonistic activity against *E. amylovora* were identified, including *Bacillus atrophaeus*, *Priestia megaterium* (previously known as *Bacillus megaterium*) and *Serratia marcescens* based on the partial 16S rDNA sequence analysis and similarity search. The plate confrontation experiments showed that strain 8 (*P. megaterium* strain KD7) had strong antagonistic activity against *E. amylovora*. The methanolic extract from cell-free supernatant of strain KD7 displayed high antibacterial activities against *E. amylovora*. Furthermore, the active compounds of strain KD7 were separated by thin layer chromatography (TLC) and the amino acids were detected by the presence of a spot with retention factor (Rf) of 0.71. Next, three lipopeptides were identified with high-resolution mass spectrometry (HRMS), including C13-surfactin [M+H]^+^ at m/z 1008.14, C15-surfactin [M+H]^+^ at m/z 1036.50, and C14-iturin A [M+H]^+^ at m/z 1043.17. Strain KD7 showed multiple antibiotic resistance, such as ampicillin, erythromycin, penicillin and tetracycline. The detached pear leaves, twigs and fruits assay showed that both protective and curative action with strain KD7 had the ability to decrease the development of fire blight. Taken together, *P. megaterium* strain KD7 is a potential effective biocontrol agent against fire blight.

## Introduction

Fire blight, caused by the bacterium *Erwinia amylovora*, is one of the most serious plant diseases that mainly affects Rosaceae, such as apple, pear, medlar and quince. The disease spreads easily and rapidly during warm and moist, especially during the bloom stages. All parts of the plant can be affected by fire blight, including leaves, shoots, flowers and fruits. The characteristic symptoms include wilt and death of flower clusters, wilting and dieback of shoots, and the affected shoots often bent into a ‘shepherd’s crook’ shape (Eastgate, 2000). Since fire blight was first reported in the United States in the 1870s, this disease has spread to more than 50 countries around the world, including countries in North America, Europe, Africa and Asia, causing enormous economic losses to fruit production (Zhao et al., 2019).

Due to the strong ability of survival and migration of *E. amylovora*, as well as high diversity of susceptible hosts, it is difficult to find a unified and effective target for eradication of *E. amylovora*, which makes fire blight difficult to control (Klee et al., 2019). Until now, the common strategy to prevention and control fire blight including pruning of infected twigs and branches, copper compounds (copper hydroxide, tribasic copper sulfate, copper sulfate basic, and copper oxychloride), and antibiotics (streptomycin, oxytetracycline, validamycin, and oxolinic acid) (Joos et al., 2014; Park et al., 2017). However, the role of copper compounds is limit because they only have protective but not curative effect against *E. amylovora*, and can cause side effects in the form of fruit russets, seriously affecting the appearance quality and commercial value (Mikiciński et al., 2019). The use of antibiotics has possible increase the risk of pathogen resistance development and cause environmental problems (McManus et al., 2002; Mikiciński et al., 2019), which has been either not permitted or prohibited in many countries.

Biological control with microbial antagonists is considered as a powerful and eco-friendly alternative in controlling fire blight (Vanneste et al., 2004). Multiple bacterium-based biological control agents have been used to control fire blight, such as *Pantoea agglomerans, Pseudomonas fluorescens*, *Rhanella aquatilis* and *Bacillus subtilis* (Chatterjee, 2001; Stockwell et al., 2010; Sharifazizi et al., 2016). Some bacteria strains have been registered or commercially available and others are in the process of registration. The most famous are *P. fluorescens* A506 (Wilson and Lindow, 1993), *P. agglomerans* P10c (Vanneste et al., 2002), *P. agglomerans* E325 (Pusey et al., 2008), *Pantoea vagans* C9-1 (Ngugi et al., 2010), *B. subtilis* QST713 (Aldwinckle et al., 2002; Bahadou et al., 2017) and BD170 (Broggini et al., 2005). However, the effectiveness of biopesticides for fire blight control was reported generally low and variable because their effects were highly influenced by environmental conditions and disease severities in each orchard, and requires combination with antibiotics (Ngugi et al., 2011). Therefore, new antagonistic bacterial strains with potential characterization and various modes of action are still need to be explored for fire blight disease control.

In recent years, several mechanisms of action have been reported to explain the suppression of *E. amylovora* by antagonistic bacteria, such as the production of secondary metabolites, nutrient competition and colonization. Various antibiotics produced by *P. agglomerans* strains including phenazine, pantocin A and B were confirmed to be effective in suppressing the fire blight pathogen (Wright et al., 2001; Giddens et al., 2002). Lipopeptides produced by *Bacillus* species have been shown to be an efficient agent against *E. amylovora* (Mora et al., 2015). *Pseudomonas* species are reported to compete with *E. amylovora* for space and nutrients (Vanneste et al., 2004; Cabrefiga et al., 2007).

In this study, bacteria from the soil of pear orchard in China were isolated, strains with antagonistic activities against *E. amylovora in vitro* were identified. Among those isolates, strain KD7 which identified as *Priestia megaterium* (formerly known as *Bacillus megaterium*) (Gupta et al., 2020) showed higher antagonist activity against *E. amylovora*, and the antibiotic substances were determined by HRMS techniques. Furthermore, the efficacy of strain KD7 for control of fire blight was evaluated on detached pear tissues. Our results will help for the effective control of fire blight.

## Materials and methods

### Bacterial strains, culture conditions, and plant material

*E. amylovora* strain 0017 which was originally isolated from pear in Kyrgyzstan was used as a reference strain. This strain was identity by polymerase chain reaction (PCR) using the primers AMS3/AMS4c, based on chromosomal *ams* gene (Kim et al., 2001). *E. amylovora* cultures were maintained and grown on nutrient agar (NA) (3.0 g/l beef extract, 5.0 g/l peptone, 15.0 g/l agar, and 5.0 g/l NaCl at pH 7.2–7.4) (Schaad et al., 2001).

Two pear varieties, ‘duli’ pear (*Pyrus betulifolia* Bunge) and Korla fragrant pear, were used to evaluate the biocontrol activity of antagonistic bacteria against *E. amylovora.* The detached leaves and twigs were selected from ‘duli’ pear, a wildtype pear widely used rootstock for grafting in China due to its resistance to biotic and abiotic stress (Song et al., 2022). The fruits were collected from Korla fragrant pear (*Pyrus sinkiangensis* Yü), a popular cultivated pear variety in China.

### Bacteria isolation and identification

The potential bacterial antagonists were isolated from the pear orchard soil in Korla of Xinjiang, China. The surface debris were removed from the soil, and soil samples were collected (5–15-cm depth) in plastic bags and stored at 4°C. Ten grams of soil sample was suspended in 90 ml of sterile distilled water (SDW), under shaken (200 rpm) for 30 min, and the 1.0 × 10^−1^ dilution was obtained. Then, the supernatant was serially diluted (1:10) in SDW, and 200 μl of the dilutions (1.0 × 10^−5^ and 1.0 × 10^−6^) were plated onto NA medium at 28°C for 24–48 h. The colonies were picked up and streaked on nutrient broth (NB) (3.0 g/l beef extract, 5.0 g/l peptone, and 5.0 g/l NaCl at pH 7.2–7.4) (Schaad et al., 2001) separately, and pure cultures were stored at 4°C for further use.

Phenotypic and biochemical traits of antagonistic bacterial isolates were tested by conventional bacteriological methods. Morphological traits such as colony color and cell motility as well as physiological fingerprints were performed on NA medium. Gram staining was performed as previously described (Ait Bahadou et al., 2018).

Genomic DNA was extracted using CTAB/NaCl method (Ashmawy et al., 2015). The bacterial isolates were identified by PCR of the 16S rDNA gene sequence using fD2 (5′- AGAGTTTGA TCCTGGCTCAG-3′) and rP1 (5’-ACGGTTACCTTGT TACGACTT-3′) primers (Weisburg et al., 1991). The PCR product was purified, sequenced, and submitted to NCBI database^1^ to identify matches with existing characterized reference sequences. A phylogenetic tree was constructed using the neighbor-joining (NJ) method in MEGA 4.0 software with 1000 bootstrap replicates (Tamura et al., 2007).

### Antimicrobial activity assays

The pathogenic bacterial cells of *E. amylovora* 0017 and antagonistic bacteria were inoculated on NB medium, respectively, and cultured at 28°C with shaking at 200 rpm. After 24 h, the bacterial suspensions were diluted to an OD_620_ of 1.0 (about 1.0 × 10^8^ to 1.0 × 10^9^ /ml) (EPPO, 2013).

The plate confrontation method was used to verify the functional activity of these isolated bacteria. 200 μl of diluted *E. amylovora* suspension was poured into an NA plate (12 cm in diameter), spread and dried at room temperature for 5 min. A single colony of potential antagonistic bacteria was picked up and spotted on the NA plate containing *E. amylovora*. After incubation (28°C, 48 h), the diameter of the inhibition zone was measured, and the antagonistic activity was determined by measuring the diameter of the inhibition zone (mm) around the isolated bacteria.

### Antibiotic resistance of *Priestia megaterium* strain KD7

The antibiotic susceptibility of strain 8, namely *P. megaterium* strain KD7, was investigated by the disk-diffusion method as described previously (Jorgensen and Ferraro, 2009). Eight commercial antimicrobial susceptibility disks (Hangzhou Microbial Reagent Co., Ltd., Hangzhou, China), each containing a defined antibiotic concentration were used for this analysis: streptomycin, kanamycin, tetracycline, erythromycin, penicillin, cefotaxime, ampicillin and ciprofloxacin. First, *P. megaterium* strain KD7 inoculum was serially diluted, and 200 μl of 1.0 × 10^−5^ dilution was spread onto 90 mm diameter NA plates. When the surface of the plate dried, each antibiotic disk was placed on the inoculated plates. Afterwards, the plates with antibiotic disks were incubated at 28°C for 24 h. The appearance of bacterial growth around the disk indicates resistant bacteria, while the inhibitory diameters around the disk indicates sensitive bacteria (Slama et al., 2019).

### Antibacterial activity of *Priestia megaterium* strain KD7 against *Erwinia amylovora in vitro*

*P. megaterium* strain KD7 was grown on NB medium in a shaking incubator at 28°C for 24 h and 200 rpm. The cultures were then centrifuged (6,000 rpm, 10 min), and the supernatant was sterilized through a 0.22 μm filter. The bacterial cells were disrupted by an ultrasonic cell breaker and filter sterilized to obtain sterile liquid inclusion.

The antibacterial activity of these two filtrates against *E. amylovora* was measured by disk-diffusion method (Zaidan et al., 2005). 10 μl of each filtrate was loaded on a sterile filter paper disk (about 6 mm diameter) and placed onto NA plate containing *E. amylovora.* As a negative control, disk was impregnated with 10 μl of SDW. After incubation (48 h, 28°C), the diameter of the inhibition zone around the disk was measured. All experiments were repeated three times independently.

### Extraction of active molecules from *Priestia megaterium* strain KD7

*P. megaterium* strain KD7 was cultured in 100 ml of NB medium for 24 h (180 rpm, 28°C). Bacterial culture was centrifuged for 10 min (6,000 rpm, 4°C), then the supernatant was collected and filtered through a 0.22 μm sterile filter.

Liquid–liquid extraction method was used to isolate antibacterial bioactive compounds from *P. megaterium* strain KD7. This method is a process of transferring compounds from one liquid phase into another based on their relative solubility in two different immiscible liquids (Huddleston et al., 1998). In this experiment, we used three different solvents (n-hexane, ethyl acetate and n-butanol) to obtain the fraction of n-hexane, ethyl acetate and n-butanol, respectively. The above cell-free supernatant was partitioned with n-hexane, ethyl acetate, and n-butanol in sequence, and the ratio of the volume of solvent and initial supernatant was 3:1. The mixture was rotated for 10 min, shaking for 30 min, and then centrifuged for 10 min at 4°C. The solvent layers were collected and dried by an evaporator.

The lipopeptide crude extracts from strain KD7 were obtained according to method previously described (Vater et al., 2002). The cell-free supernatant was adjusted to pH 2.0 with 6 M HCl and stored at 4°C overnight to precipitate the lipopeptides. After centrifugation (6,000 rpm, 10 min), the extracted lipopeptides were dissolved in methanol for *in vitro* evaluating antimicrobial activity.

The antibacterial activity of extracts was evaluated using the disk-diffusion method. 10 μl of each extract was loaded onto a sterile filter paper disk (6 mm diameter), then placed on NA plate containing *E. amylovora* and incubated at 28°C for 24–48 h. Antibacterial activity was determined by measuring the zone of inhibition around the disks.

### Thin layer chromatography

The antibacterial components of the extracted supernatant were detected by thin layer chromatography (TLC) (Batrakov et al., 2003). A chloroform-methanol–water mixture (65: 25: 4, v/v/v) was used for the separation, and the spot was visualized by spraying TLC plate with ninhydrin solution. The properties of the separated molecules were determined by calculating the retention factor (Rf) values. The experiments were repeated three times.

### Mass spectrometry analysis of antibacterial components

To identify the substances produced by strain KD7, the crude methanol extracts were analyzed by High-resolution mass spectral (HRMS) on an Orbitrap Exploris 480 mass spectrometer and EASY-nLC liquid chromatography system (Thermo Fisher Scientific) equipped with an electrospray ionization (ESI) in positive mode. The ion source was set up as follows: capillary voltage: 2300 V, ion transfer tube temperature: 320°C. Detection was performed with resolution of 120,000, scan range from 200 to 1,200 m/z, 1.6 m/z isolation window. Separation of compounds was performed on ChromXP C_18_ column (3 μm, 250 mm × 75 μm, Eksigent) using H_2_O (A)/acetonitrile (B) both containing 0.1% HCOOH as a mobile phase. The injection volume was 2 μl and the flow rate was 0.3 ml min^−1^.

### Evaluation of antagonistic activity of *Priestia megaterium* strain KD7 in detached pear tissues

Under laboratory conditions, the potential protective and curative effect of *P. megaterium* strain KD7 against *E. amylovora* was investigated on detached pear tissues: leaves and twigs of ‘duli’ pear, and fruits of Korla fragrant pear. The streptomycin was used to evaluate the efficiency of the treatment.

To evaluate the antimicrobial effects of strain KD7 on leaves, the detached leaves of ‘duli’ pear were washed with tap water, disinfected by immersion in 3% bleach for 10 min, and then washed with SDW three times. For negative control, leaves were injected with SDW (100 μl) into the intercellular spaces on the hollow petiole of the mature leaves using a syringe and then sprayed with SDW to keep all the leaves in the same conditions (Vanneste et al., 2004; Medhioub et al., 2022). The same thing was repeated for positive control by injecting 100 μl suspensions of pathogenic bacteria *E. amylovora* 0017 (1.0 × 10^8^ CFU/ml) instead of SDW. After 48 h of incubation, leaves were sprayed with suspensions of antagonistic bacteria strain KD7 (1.0 × 10^8^ CFU/ml) and streptomycin solution, separately. For protective tests, leaves were sprayed with strain KD7 or streptomycin, separately. After 48 h, leaves were injected with 100 μl pathogenic bacterial suspensions. For curative tests, leaves were treated inversely to the protective treatments. To observe the effect of strain KD7 in the leaves, a treatment control was designed as follows: leaves were sprayed with suspensions of strain KD7. After 48 h, the suspensions of strain KD7 were sprayed on the leaves again. The treated leaves were incubated in a climate chamber (28°C, 12:12 L:D) and symptoms were assessed from 3 to 15 days of post-inoculation (dpi). Three leaves were used for each treatment and all experiments were repeated three times. Disease severity was assessed by evaluating the percentage of leaf area affected (Vincelli and Hershman, 2011). The scale from level 1–13 was used to evaluate foliar necrosis index.

To investigate the antimicrobial effects of strain KD7 on twigs, healthy detached twigs of ‘duli’ pear were cut into equal-sized pieces (1.5 cm diameter and 20 cm long) and surface sterilized as described above. Each twig was artificial injured before treatment. For negative control, twigs were sprayed with SDW. For positive control, twigs were sprayed with suspensions of pathogenic bacterial (1.0 × 10^8^ CFU/ml). For protective tests, twigs were sprayed with suspensions of antagonistic bacteria strain KD7 (1.0 × 10^8^ CFU/ml) and streptomycin, separately. After 48 h, twigs were sprayed with suspensions of pathogenic bacterial (1.0 × 10^8^ CFU/ml). For curative tests, twigs treated reversely to the protective tests. Three twigs were used for each treatment with three independent experiments. All twigs were incubated at 28°C in darkness for a period of 7 to 15 days. After 15 dpi, disease severity was assessed by measuring the necrosis at the inoculation site based on the vascular browning index of twigs (0–4 scale): 0 = absence of browning, 1 = 1–10% browning, 2 = 11–25% browning, 3 = 26–75% browning, and 4 = 76–100% browning (Medhioub et al., 2022).

To evaluate the antimicrobial effects of strain KD7 on fruits, Korla fragrant pear fruits were surface sterilized and air dried under filter-sterilized air flow. For negative control, three small holes (2 mm wide, 5 mm depth) were made on each fruit and 100 μl of SDW was injected on each hole. For positive control, 100 μl pathogenic bacterial suspension (1.0 × 10^8^ CFU/ml) was injected instead of SDW. After 48 h, wounded fruits were sprayed with strain KD7 (1.0 × 10^8^ CFU/ml) and streptomycin solution, separately. For protective tests, fruits were sprayed with antagonistic bacteria strain KD7 (1.0 × 10^8^ CFU/ml) and streptomycin, separately. After 48 h, wounded fruits were sprayed with suspensions of pathogenic bacterial (1.0 × 10^8^ CFU/ml). For curative tests, fruits treated reversely to the protective treatments. Symptoms were recorded from 6 dpi to 15 dpi. Disease severity was assessed based on a fruit infection index (0–4 scale): 0 = no necrotic spots, 1 = necrotic spots of 1–5 mm in diameter, 2 = necrotic spots of 6–10 mm in diameter, 3 = necrotic spots of 11–20 mm in diameter, and 4 = necrotic spots over 21–30 mm in diameter (Medhioub et al., 2022).

### Statistical analysis

SPSS 17.0 and GraphPad Prism 7 were used for statistical analysis and mapping of experimental data, and variance analysis was performed by LSD multiple comparison method (*p* ≤ 0.05).

## Results

### Isolation and identification of antagonistic bacteria strains

**Figure 1:**
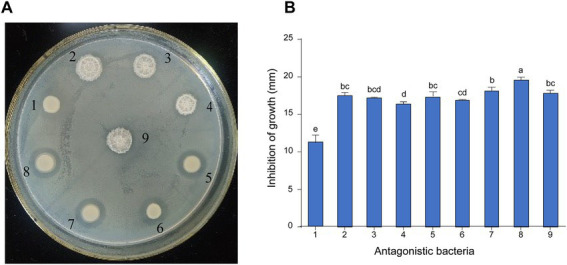
Screening and inhibition of *E. amylovora* by antagonistic bacteria. **(A)** The effect of antagonistic bacteria against *E. amylovora*. (1) *Serratia marcescens* D6; (2) *Bacillus atrophaeus* B4; (3) *B. atrophaeus* KB16; (4) *B. atrophaeus* KB14; (5) *Priestia megaterium* KB3; (6) *S. marcescens* D5; (7) *P. megaterium* KB17; (8) *P. megaterium* KD7; (9) *B. atrophaeus* B8. **(B)** Diameter of antagonistic inhibition zone of nine strains. Data with the same letters are not significantly different at *p* ≤ 0.05.

A total of 16 bacterial strains were isolated from pear orchard soil of infected pear trees, among which nine strains showed effective antagonistic activity against *E. amylovora* using the plate confrontation method (Figure 1A). The diameter of the inhibition zones of the antagonistic bacteria against *E. amylovora* varied from 11.17 ± 0.60 to 19.57 ± 0.23 mm. Among them, strain 8 exhibited stronger inhibition activities against *E. amylovora*, with the diameter of the inhibition zone reaching 19.57 ± 0.23 mm (Figure 1B), which was larger than that of other antagonistic bacteria. Thus, strain 8 was selected for further characterization and identification.

Table 1 summarizes morphological, physiological and biochemical traits of nine antagonistic bacteria. Results of Gram staining showed that seven antagonistic bacteria (2, 3, 4, 5, 7, 8 and 9) were Gram-positive, rod-shaped producing white colonies. Furthermore, all bacterial isolates were motile, and showed positive to glucose, saccharose, catalase, gelatin liquefaction and starch hydrolysis. All isolates showed MR reaction negative, while three isolates (5, 7 and 8) showed V-P test negative. According to these phenotypic fingerprints, the selected putative antagonistic bacteria against *E. amylovora* were grouped into the following genera: *Bacillus* (2, 3, 4, and 9), *Priestia* (5, 7, and 8) and *Serratia* (1 and 6).

**Table 1:** Morphological, physiological and biochemical characters of nine antagonistic bacteria.

Bacterial isolates	1	2	3	4	5	6	7	8	9
Shape	Rod	Rod	Rod	Rod	Rod	Rod	Rod	Rod	Rod
Motility	+	+	+	+	+	+	+	+	+
Color of colony	White	White	White	White	White	White	White	White	White
Gram Staining	−	+	+	+	+	−	+	+	+
Gelatin liquefaction	+	+	+	+	+	+	+	+	+
Starch hydrolysis	+	+	+	+	+	+	+	+	+
MR reaction	−	−	−	−	−	−	−	−	−
V-P test	+	+	+	+	−	+	−	−	+
Catalase	+	+	+	+	+	+	+	+	+
Fermentation of glucose	+	+	+	+	+	+	+	+	+
Fermentation of sucrose	+	+	+	+	+	+	+	+	+

“+” and “−” represent positive and negative reactions, respectively.

**Figure 2:**
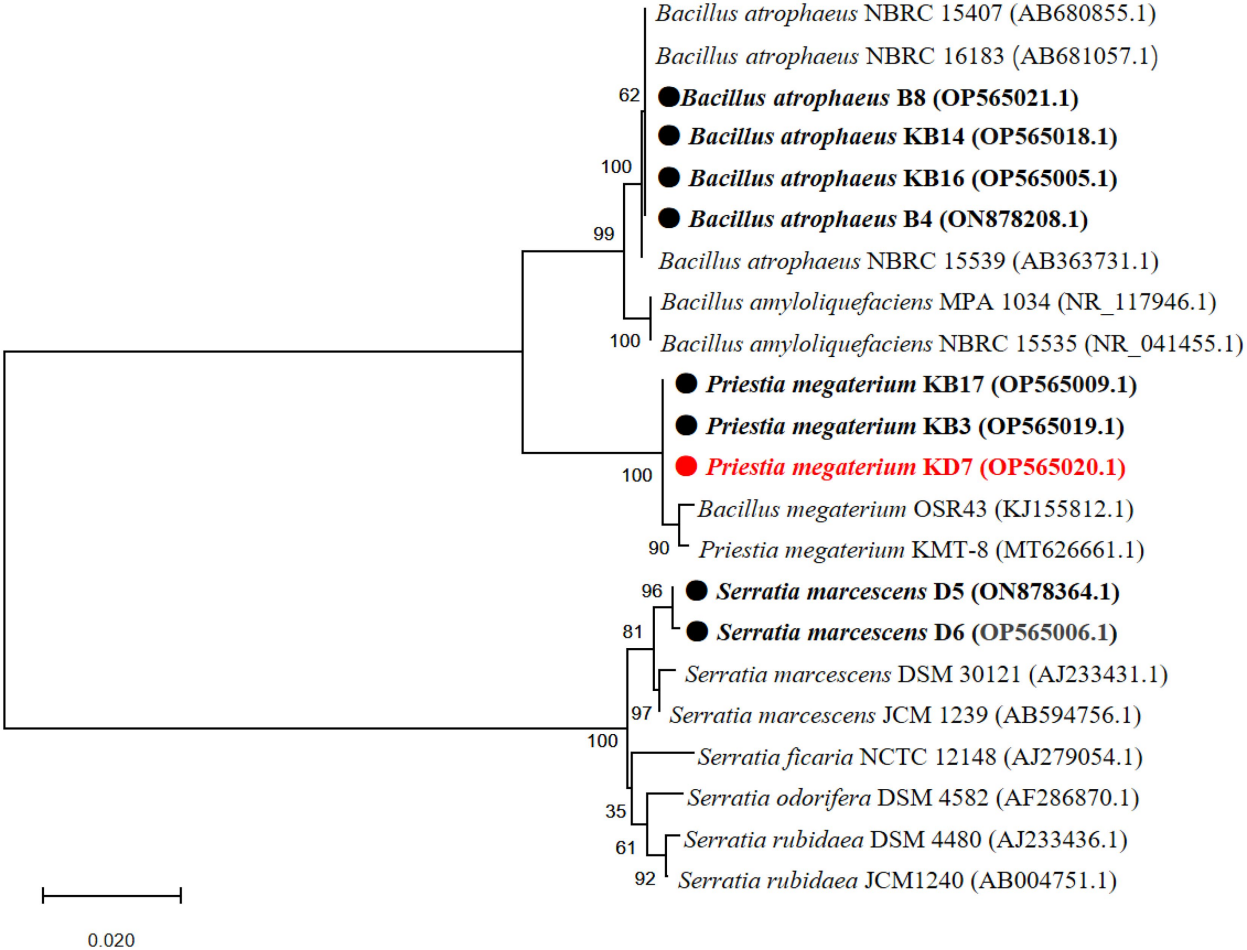
Phylogenetic tree of nine antagonistic bacteria isolates with related species on the basis of partial 16S rDNA sequences. The analysis was conducted with MEGA 4.0 using neighbor-joining method with 1,000 bootstrap replicates.

A phylogenetic tree based on partial 16S rDNA sequence of isolated strains and other bacterial species from the GenBank database was constructed. As shown in Figure 2, strain 2, 3, 4 and 9 shared the highest sequence similarity (>99%) with *Bacillus atrophaeus* (AB680855.1, AB681057.1 and AB363731.1), which was identified as *B. atrophaeus*. Strain 5, 7 and 8 shared the highest sequence similarity (>99%) with *B. megaterium* (KJ155812.1 and MT626661.1), which was identified as *P. megaterium*. Strain 1 and 6 shared the highest sequence similarity (>99%) with *Serratia marcescens* (AJ233431.1 and AB594756.1), which was identified as *S. marcescens*. The 16S rDNA sequences of the 9 isolated strains were submitted to the NCBI for GenBank accession numbers as follows: strain 1 (*S. marcescens* D6, OP565006.1), strain 2 (*B. atrophaeus* B4, ON878208.1), strain 3 (*B. atrophaeus* KB16, OP565005.1), strain 4 (*B. atrophaeus* KB14, OP565018.1), strain 5 (*P. megaterium* KB3, OP565019.1), strain 6 (*S. marcescens* D5, ON878364.1), strain 7 (*P. megaterium* KB17, OP565009.1), strain 8 (*P. megaterium* KD7, OP565020.1), and strain 9 (*B. atrophaeus* B8, OP565021.1).

### Antibacterial activity of *Priestia megaterium* strain KD7

**Figure 3:**
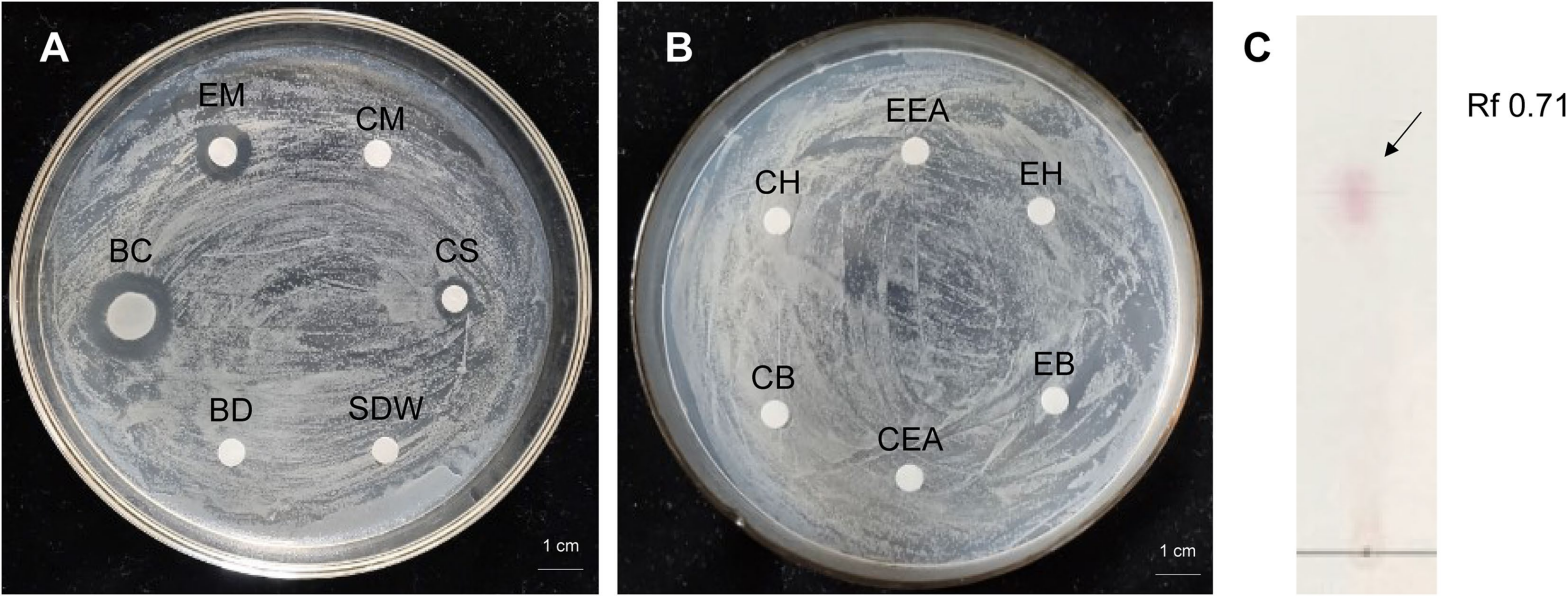
The antibacterial activity analysis of organic extracts from *P. megaterium* strain KD7 against *Erwinia amylovora* on NA medium **(A,B)**, and separation of active compounds by thin layer chromatography (TLC) **(C)**. EM, extracted with methanol; CM, methanol control; BC, bacteria culture broth (1.0 × 10^8^ CFU/ml); CS, filtrate from the bacterial cell-free supernatant; BD, filtrate from the bacterial cell disruption; EH, extracted with n-hexane; CH, n-hexane control; EB, extracted with n-butanol; CB, n-butanol control; EEA, extracted with ethyl acetate; CEA, ethyl acetate control; SDW, sterile distilled water (control).

Antibacterial activity of strain 8 (*P. megaterium* KD7) against *E. amylovora* was checked by disk-diffusion method. The cell-free supernatant (CS) of strain KD7 exhibited antimicrobial activity compared to the bacterial cell disruption (BD) (Figure 3A), indicating that the extracellular secondary metabolites secreted by strain KD7 contribute to the antibacterial activity. The growth inhibition activity against *E. amylovora* was detected by the four extracts from strain KD7, which were the extract with methanol (EM), n-hexane (EH), ethyl acetate (EEA) and n-butanol (EB). After incubation, only the bacteria culture broth (BC) and methanol extracts (EM) exhibited an antibacterial activity (Figures 3A,B). The methanol extract was further detected by TLC analysis, which showed a spot with Rf values 0.71 after ninhydrin staining (Figure 3C), suggesting the presence of amino acids in the methanol extract.

### HRMS (ESI) analysis of lipopeptides

Using the HRMS(ESI) method, two types of lipopeptides were identified from the crude methanol extracts of strain KD7, including surfactin and iturin A (Table 2; Supplementary Figure S1). From the HRMS results, the major products of m/z 1008.14, and 1036.50 were identified as C13-surfactin [M + H]^+^ and C15-surfactin [M + H]^+^, respectively, and the major product of m/z 1043.17 assigned to C14-iturin A [M + H]^+^ (Kalai-Grami et al., 2016; Wang et al., 2022). The results revealed that surfactin and iturin A played important roles in the antibacterial activities of strain KD7.

**Table 2:** HRMS (ESI) analysis of antagonistic lipopeptides from the crude extracts of strain KD7.

m/z	Lipopeptide assignment
1008.14	C13 surfactin [M + H]^+^
1036.50	C15 surfactin [M + H]^+^
1043.17	C14 iturin A [M + H]^+^

### Antibiotic resistance of *Priestia megaterium* strain KD7

**Figure 4:**
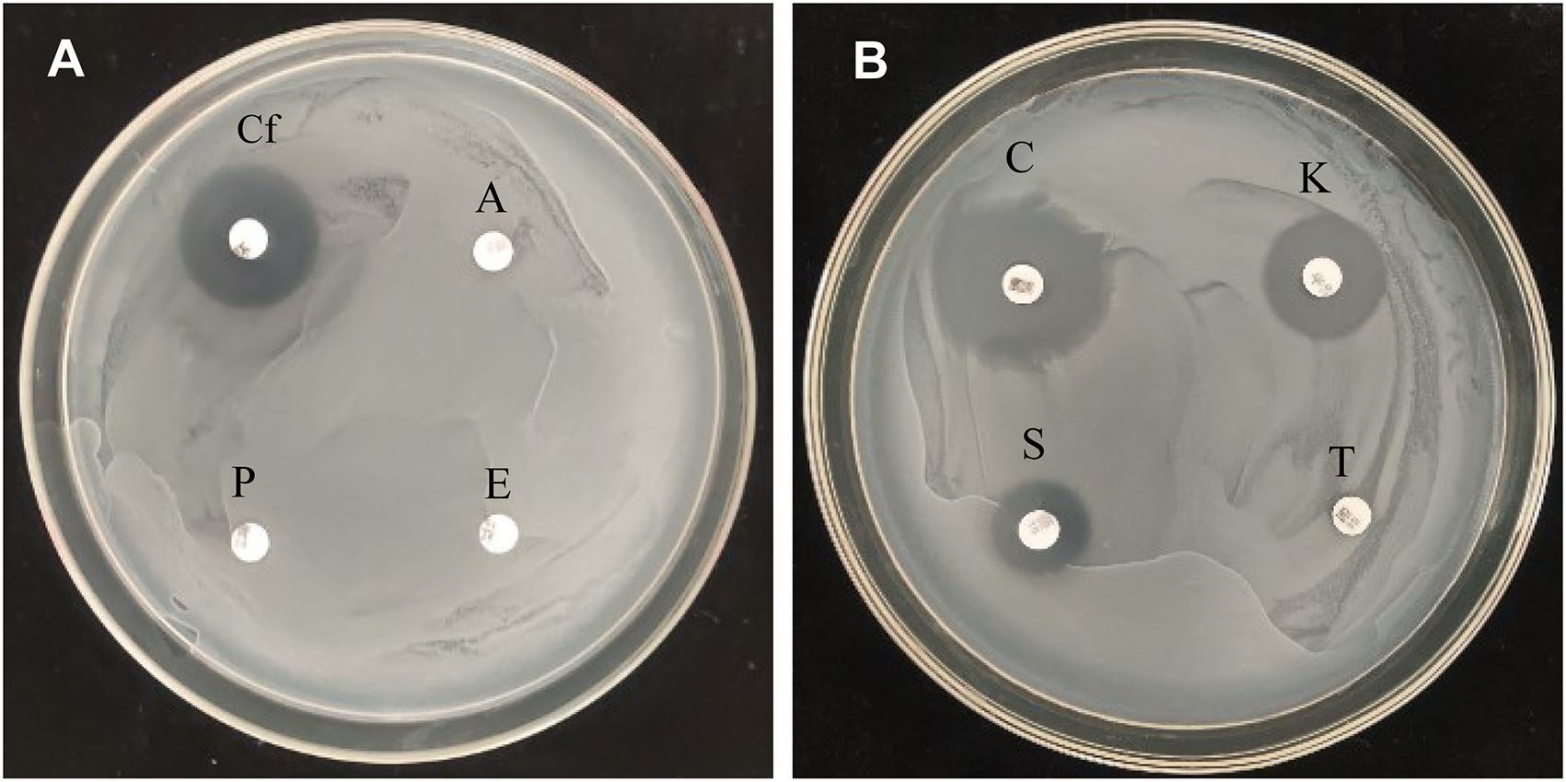
Antibiotic sensitivity test of *P. megaterium* KD7. **(A)** *Cf*, ciprofloxacin; A, ampicillin; E, erythromycin; P, penicillin. **(B)** C, cefotaxim; K, kanamycin; T, tetracycline; S, streptomycin.

Antibiotic resistance of *P. megaterium* strain KD7 was tested using disk-diffusion method. As shown in Figure 4, strain KD7 was resistant to ampicillin, erythromycin, penicillin and tetracycline, and susceptible to ciprofloxacin, cefotaxim, kanamycin and streptomycin.

### Effectiveness on detached pear tissues

**Figure 5:**
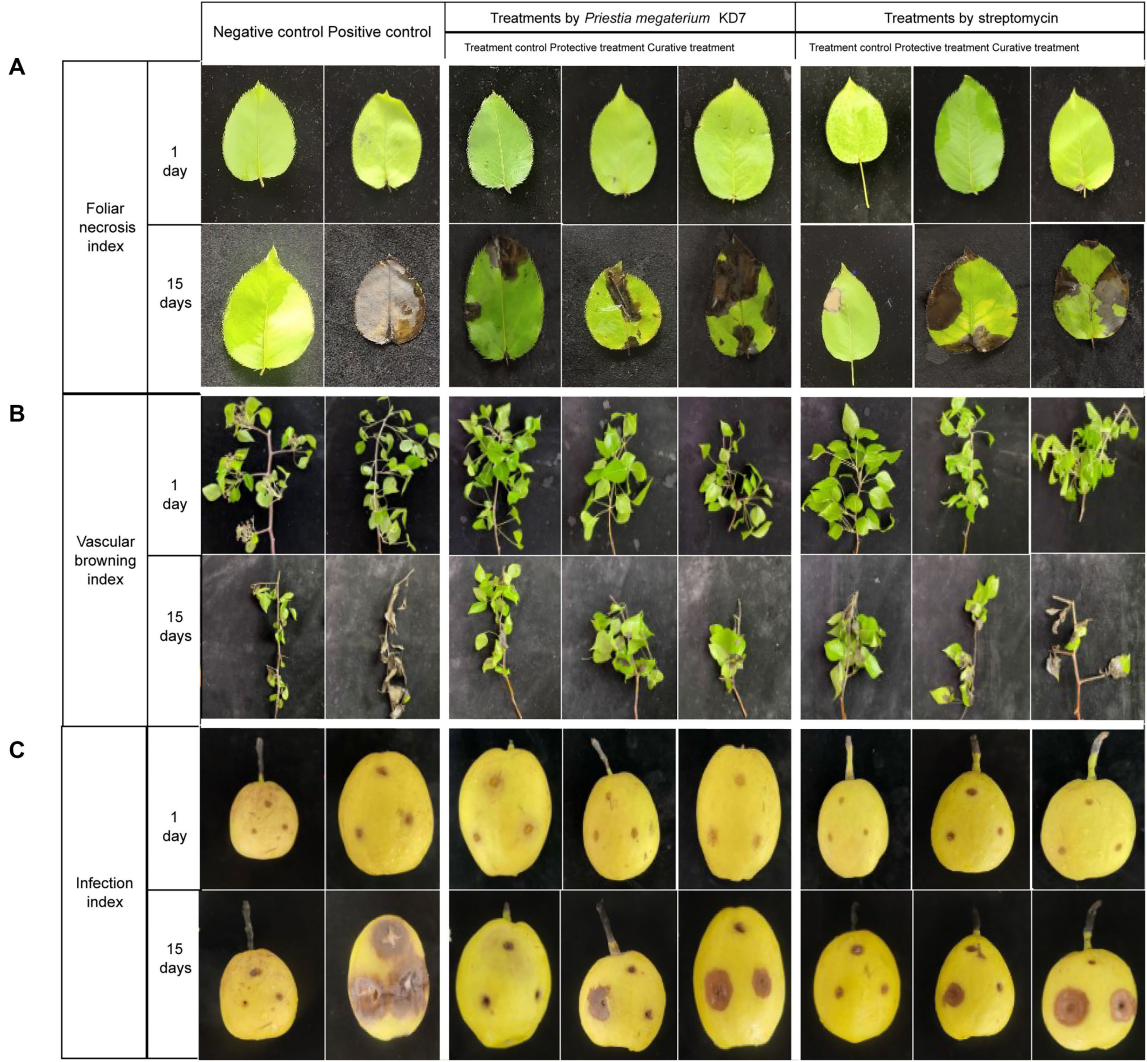
Protective and curative effect of *P. megaterium* strain KD7 against *Erwinia amylovora* on detached pear leaves **(A)**, twigs **(B)** and fruits **(C)** after 15 days post-inoculation (dpi). The leaves and twigs were collected from wildtype ‘duli’ pear, and fruits from Korla fragrant pear.

**Figure 6:**
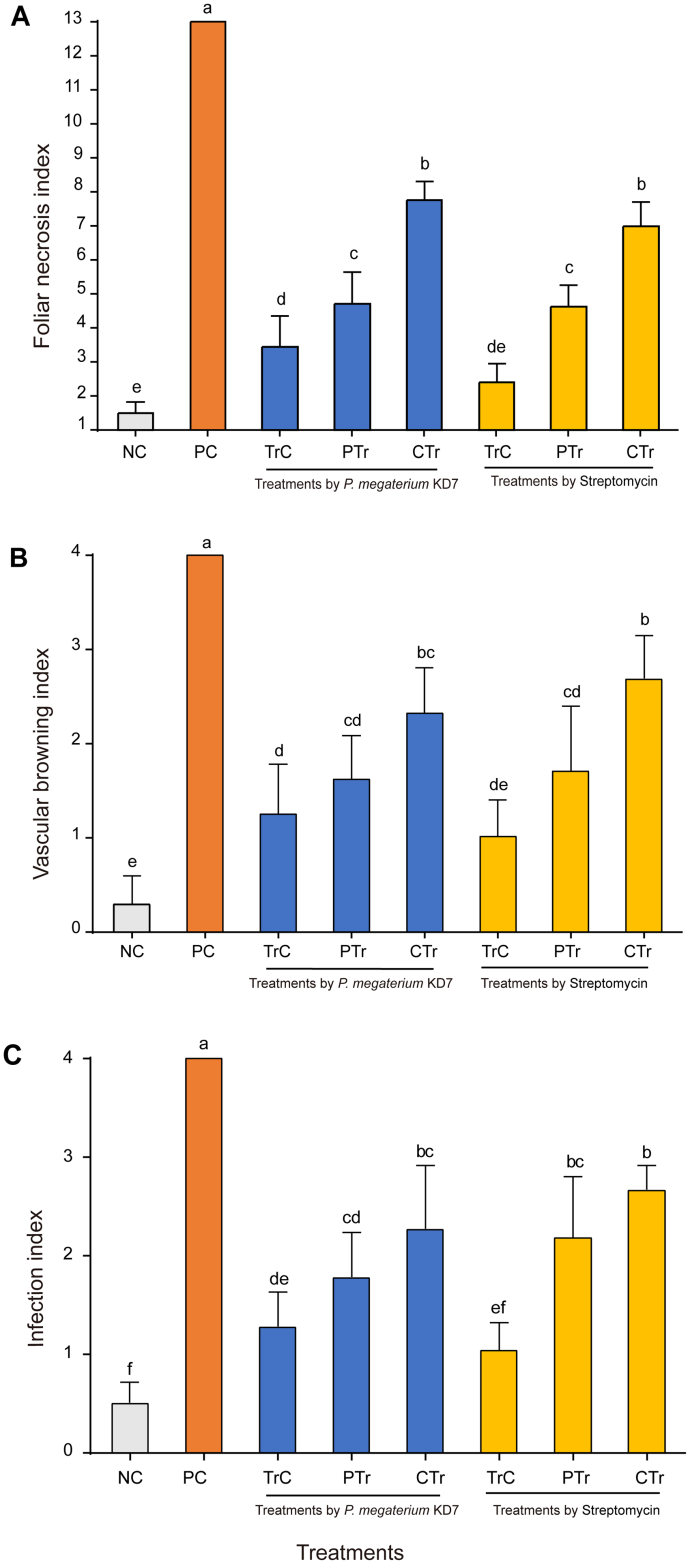
The disease index of fire blight in detached pear tissues treated with *P. megaterium* strain KD7 after 15 days post-inoculation (dpi): **(A)** leaves, **(B)** twigs and **(C)** fruits. NC, negative control; PC, positive control; TrC, treatment control; PTr, protective treatment; CTr, curative treatment. Means with the same letter are not significantly different at *p* ≤ 0.05.

Detached pear leaves, twigs and fruits were used to assay the biocontrol efficacy of strain KD7 against *E. amylovora*. In detached ‘duli’ pear leaves assay (Figures 5A, 6A), curative treatments with strain KD7 reduced the disease severity significantly compared to the control. In the positive control group, necrosis occurred by 3 dpi, exudates released and necrotic spots appeared from the wound site of leaf on the 6th dpi. From 9 to 15 dpi, the remaining leaves were affected rapidly with a foliar necrosis index of 13.00 ± 0.00. By contrast, a small partial of strain KD7-treated leaves appeared disease symptoms, with the foliar necrosis index of 4.72 ± 0.53 and 7.77 ± 0.30 for protective and curative treatment, respectively. Comparatively, foliar necrosis index was 4.74 ± 0.44 and 6.99 ± 0.41 for protective and curative treatment with streptomycin, respectively. The results indicated that strain KD7 exhibited good biological control potential against *E. amylovora*.

In the second experiment on ‘duli’ pear twigs (Figures 5B, 6B), no necrosis was observed in water-treated twigs (negative control) at 15 dpi. In contrast, the first symptoms began to appear at 3 dpi on *E. amylovora* treated twigs (positive control), and reached the disease incidence by 100% at 15 dpi. Compared with positive control (index = 4.00), vascular browning index was reduced significantly in strain KD7-treated twigs in protective and curative treatments (index = 1.62 ± 0.27 and 2.32 ± 0.28 respectively), which was similar as streptomycin-treated twigs in protective and curative treatments (index = 1.71 ± 0.40 and 2.69 ± 0.27 respectively).

The third experiment on the fruits of Korla fragrant pear (Figures 5C, 6C), positive control inoculated with *E. amylovora* appeared necrosis after 6 dpi, and reached the disease incidence of 100% (index = 4.00) at 15 dpi. However, the infection index was significantly reduced both in both protective and curative treatments with strain KD7 (index = 1.78 ± 0.26 and 2.27 ± 0.37, respectively), indicating that strain KD7 decreased the pathogen development compared with positive control.

In all *in vivo* assays, disease symptoms and pathogen development in pear tissues treated with strain KD7 were significantly lower than positive control (*p* < 0.05). Furthermore, the effectiveness of protective and curative treatments of strain KD7 against *E. amylovora* were similar to those of streptomycin, indicating its potential biological control agent for controlling fire blight.

## Discussion

In recent years, biological control of plant diseases has received increasing attention due to the urgent need to find the eco-friendly alternatives to chemical pesticides (Ongena and Jacques, 2008). In this study, we isolated bacterial antagonists and investigated the inhibition effects against *E. amylovora in vitro* and *in vivo*. A total of 9 bacteria with stable antagonistic against *E. amylovora* were isolated from pear orchard soil. These isolates were identified as *B. atrophaeus*, *P. megaterium* and *S. marcescens*. Among the isolated bacteria, strain 8 (*P. megaterium* strain KD7) showed the highest inhibition effect against *E. amylovora in vitro* experiments. *P. megaterium* is a key microorganism for the biological control of plant diseases which can enhance host plants defenses against diverse pathogens, such as *Septoria tritici* blotch in wheat (Kildea et al., 2008), *Aspergillus flavus* in rice (Mannaa and Kim, 2018). However, little is known about the role of *P. megaterium* in inhibiting fire blight pathogen. Our results showed that the strain KD7 of *P. megaterium* was effective against *E. amylovora* in detached pear tissues, indicating it as a potential biological control agent to control fire blight disease.

It has been shown that *Bacillus* genus against plant pathogens due to the production of antimicrobial compounds (Ariza and Sánchez, 2012). Most important bioactive molecules produced by *Bacillus* genus are non-ribosomal peptides and lipopeptides, polyketides, siderophore and bacteriocins (Fira et al., 2018). *Bacillus* lipopeptides include three families: fengycin, surfactin and iturin (Ongena and Jacques, 2008; Dimkić et al., 2017). The iturins have shown strong antifungal activities against a wide variety of yeast and filamentous fungi but have limited antibacterial activities; The fengycins have strong antifungal activities, especially on filamentous fungi, and their antibacterial activities have been reported recently (Villegas-Escobar et al., 2018); Surfactins have significant bactericidal activities (Zhou et al., 2012; Chen et al., 2020). The molecular mechanisms of disease control for lipopeptides include direct *via* interact with the biological membrane of bacterial and fungal pathogens (Patel et al., 2011), and indirect *via* induction of systemic resistance in plant (Jourdan et al., 2009; Falardeau et al., 2013). *P. megaterium* strains produce a broad spectrum of bioactive lipopeptides, including surfactins, lichenysin, iturin A, fengycins A and B (Pueyo et al., 2009). In this study, both the cell-free supernatant and the methanol extracts from *P. megaterium* strain KD7 *in vitro* showed antibacterial activities against *E. amylovora,* indicating that the bioactive compounds are more likely extracellular secondary metabolites and that these metabolites are hydrophobic, which is the characteristics of *Bacillus* lipopeptides (Romero et al., 2007). Further TLC assay confirmed that the methanolic extracts contained amino acids. Our findings are in accordance with previous studies (Mohammad et al., 2018; Medhioub et al., 2022). The major spot was observed with Rf value of 0.71, suggesting the presence of lipopeptides in the fraction. Similar Rf value (Rf = 0.7) was reported for surfactin lipopeptide by Romero et al. (2007) and Jakinala et al. (2019). Next, two lipopeptides families were identified with HRMS(ESI) analysis, including surfactin and iturin A, indicating that strain KD7 secreted these lipopeptides responsible for antibacterial activities against *E. amylovora*.

Many bacteria have acquired resistance to some common antibiotics and give them the ability to survive antibiotic treatment. So, antibiotics sensitivity evaluation is important in determining if bacteria are resistant to certain antibiotics. Streptomycin is the most effective and widely used chemical control in many countries for fire blight. Previous study (Patel et al., 2017) demonstrated that combining biocontrol agents and antibiotics are non-compatible in biocontrol strategies. In this study, strain KD7 had high levels of multi-resistance to antibiotics, but sensitive to streptomycin, indicated that strain KD7 and streptomycin were non-compatible and should be applied separately in fire blight control.

In recent years, several authors reported the effect of bacterial antagonists against *E. amylovora* on detached plant tissues. *Lactobacillus plantarum* strains have been demonstrated to be effective in controlling fire blight on pear leaves, flowers and fruits, as well as in whole plants (Roselló et al., 2013). *Pantoea agglomerans* P10c and *B. subtilis* QST713 significantly reduced the incidence of fire blight on detached pear blossoms, and reduced blossom infection under field conditions (Bahadou et al., 2017). The bacterial antagonists (*Pseudomonas vancouverensis* L16, *P. congelans* 35 M, and *Enterobacter ludwigii* 43 M) had high protective ability on apple branches, blossoms and shoots, and even more effective than the copper product (Mikiciński et al., 2019). In this study, *P. megaterium* strain KD7 exhibited significant inhibitory activity against *E. amylovora* on detached pear leaves, twigs and fruits. However, further studies are needed to investigate the efficacy of strain KD7 against fire blight disease under natural environmental conditions.

## Conclusion

From the soil environment of pear orchard in China 16 microbial isolates were obtained. By plate confrontation method, some *Priestia*, *Bacillus* and *Serratia* species revealed inhibitory effect against *E. amylovora*. The strain 8 (*P. megaterium* strain KD7) was evaluated *in vitro* and *in vivo* study on detached pear tissues, which is a potential source of a new biocontrol agent to control fire blight.

## Data availability statement

The original contributions presented in the study are included in the article/Supplementary material, further inquiries can be directed to the corresponding author/s.

## Author contributions

ZC, LS, and LH designed the work. ZC and LH performed the experiments. WM, LZ, and DG analyzed the data. LS and ZC wrote and revised the manuscript. All authors contributed to the article and approved the submitted version.

## Funding

This work was supported by College of Life Science, Shihezi University, China.

## Conflict of interest

The authors declare that the research was conducted in the absence of any commercial or financial relationships that could be construed as a potential conflict of interest.

## Publisher’s note

All claims expressed in this article are solely those of the authors and do not necessarily represent those of their affiliated organizations, or those of the publisher, the editors and the reviewers. Any product that may be evaluated in this article, or claim that may be made by its manufacturer, is not guaranteed or endorsed by the publisher.

## Footnotes

^1^https://blast.ncbi.nlm.nih.gov/

## Supplementary material

SUPPLEMENTARY FIGURE S1 HRMS (ESI) analysis of the crude methanolic extract from strain KD7. **(A)** surfactin and **(B)** iturin A.
