# Supplementary figures and images for: Isolation and characterization of Priestia megaterium KD7 for the biological control of pear fire blight
Source: Front Microbiol. 2023 Mar 9;14:1099664. doi: 10.3389/fmicb.2023.1099664 (PMC10033528; doi:10.3389/fmicb.2023.1099664)

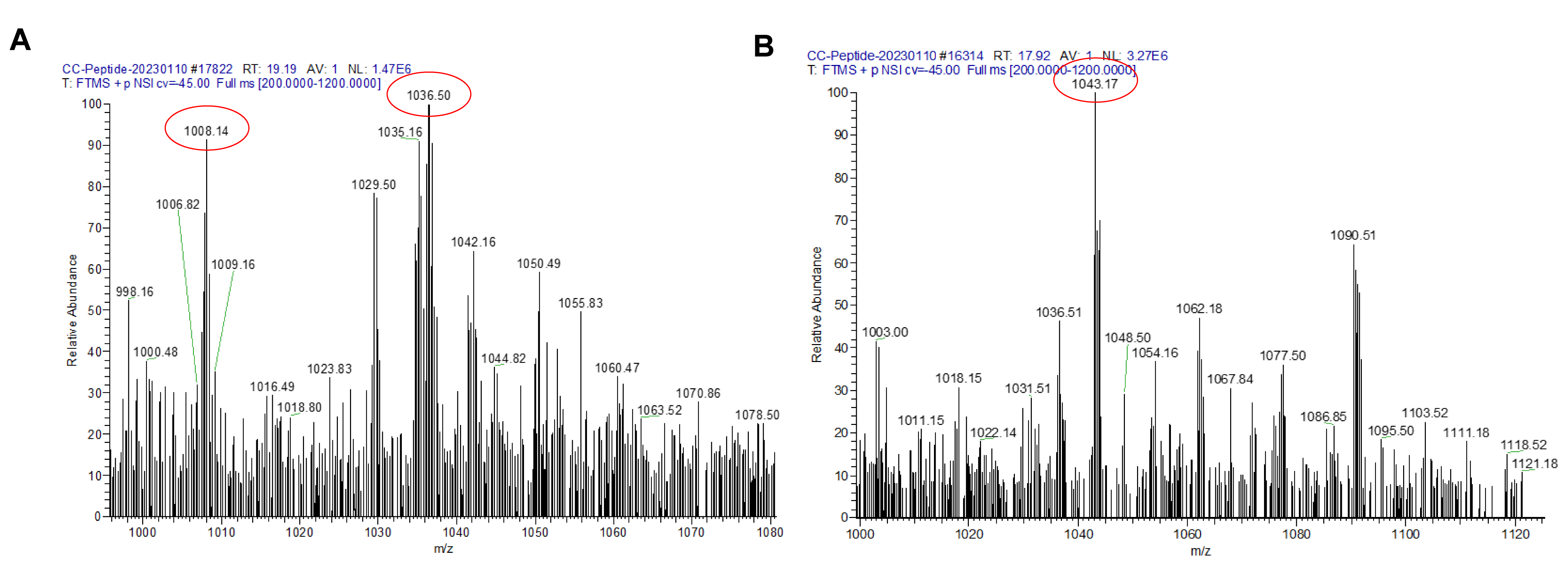

Supplement: SUPPLEMENTARY FIGURE S1 — HRMS (ESI) analysis of the crude methanolic extract from strain KD7. (A) surfactin and (B) iturin A. [file Image_1.TIF]
